# Extent of Linkage Disequilibrium and Effective Population Size in Four South African Sanga Cattle Breeds

**DOI:** 10.3389/fgene.2015.00337

**Published:** 2015-12-01

**Authors:** Sithembile O. Makina, Jeremy F. Taylor, Este van Marle-Köster, Farai C. Muchadeyi, Mahlako L. Makgahlela, Michael D. MacNeil, Azwihangwisi Maiwashe

**Affiliations:** ^1^Agricultural Research Council-Animal Production InstitutePretoria, South Africa; ^2^Department of Animal and Wildlife Sciences, University of PretoriaPretoria, South Africa; ^3^Division of Animal Sciences, University of MissouriColumbia, MO, USA; ^4^Agricultural Research Council-Biotechnology PlatformPretoria, South Africa; ^5^Department of Animal, Wildlife and Grassland Sciences, University of Free StateBloemfontein, South Africa; ^6^Delta GMiles City, MT, USA

**Keywords:** linkage disequilibrium, effective population size, persistence of phase, cattle breeds

## Abstract

Knowledge on the extent of linkage disequilibrium (LD) in livestock populations is essential to determine the minimum distance between markers required for effective coverage when conducting genome-wide association studies (GWAS). This study evaluated the extent of LD, persistence of allelic phase and effective population size (Ne) for four Sanga cattle breeds in South Africa including the Afrikaner (*n* = 44), Nguni (*n* = 54), Drakensberger (*n* = 47), and Bonsmara breeds (*n* = 46), using Angus (*n* = 31) and Holstein (*n* = 29) as reference populations. We found that moderate LD extends up to inter-marker distances of 40–60 kb in Angus (0.21) and Holstein (0.21) and up to 100 kb in Afrikaner (0.20). This suggests that genomic selection and association studies performed within these breeds using an average inter-marker *r*^2^≥ 0.20 would require about 30,000–50,000 SNPs. However, *r*^2^≥ 0.20 extended only up to 10–20 kb in the Nguni and Drakensberger and 20–40 kb in the Bonsmara indicating that 75,000 to 150,000 SNPs would be necessary for GWAS in these breeds. Correlation between alleles at contiguous loci indicated that phase was not strongly preserved between breeds. This suggests the need for breed-specific reference populations in which a much greater density of markers should be scored to identify breed specific haplotypes which may then be imputed into multi-breed commercial populations. Analysis of effective population size based on the extent of LD, revealed Ne = 95 (Nguni), Ne = 87 (Drakensberger), Ne = 77 (Bonsmara), and Ne = 41 (Afrikaner). Results of this study form the basis for implementation of genomic selection programs in the Sanga breeds of South Africa.

## Background

Conventional selection programs based on quantitative principles have worked remarkably well and have allowed for significant genetic progress in South African beef cattle breeds over many decades (Scholtz, 2010). However, more effective selection and breeding strategies may result from the incorporation of molecular information (Goddard and Hayes, 2007). This may be achieved through marker assisted selection (MAS) or genomic selection (Meuwissen et al., 2001). Marker-assisted selection in livestock breeding programs relies on linkage between quantitative trait loci (QTL) and genetic markers, whereas genomic selection relies on the estimation of identity by descent using markers to establish identity by state. Markers can be directly causal for genetic variation, or may be in linkage equilibrium (LE) or linkage disequilibrium (LD) with causal variants (Dekkers, 2004). Causal markers have historically been very difficult to discover and it has been even more challenging to prove functional causality (Dekkers, 2010). Markers found to be in linkage with causal variants within families, but in LE in the population as a whole are not effective for use in MAS (Goddard and Hayes, 2007; Dekkers, 2010). In contrast, markers in LD with causal variants provide an alternative approach to the implementation of MAS in livestock populations and may be discovered in genome-wide association studies (GWAS) by searching for genomic regions that are associated with traits of economic importance (Goddard and Hayes, 2007). Discovery of these regions has been enabled by advances in genomic technologies, including sequencing of whole genomes of livestock species that include cattle, chickens, sheep, and goats (Hayes et al., 2009).

Successful application of LD information in different populations requires significant population-wide disequilibrium between markers and a large-effect QTL in order that marker alleles can predict the QTL alleles in individuals sampled from across the entire population (Hayes et al., 2009). McKay et al. (2007) assessed the extent of LD in cattle using 2670 single nucleotide polymorphism (SNP) markers genotyped in eight breeds from *Bos taurus* and *Bos indicus* cattle. Results from their study revealed that moderate LD (*r*^2^ = 0.20) extended to 40–60 kb in cattle, which indicated that about 50,000 SNPs would capture most of the LD information necessary for GWAS in European *B. taurus* breeds. In another study, Thévenon et al. (2007) evaluated the extent of LD in *n* = 363 *B. indicus* × *B. taurus* cattle from western Africa using 42 microsatellite markers on chromosomes 1, 4, and 7. Their results indicated that LD extended for shorter distances than had previously been observed in cattle from developed countries. This indicated that GWAS performed in these hybrids would require from75,000 to 300,000 SNP markers. Edea et al. (2014) evaluated the extent of LD in the indigenous Zebu and Sanga breeds of Ethiopia using the Illumina BovineSNP50 BeadChip (Matukumalli et al., 2009) and found that the extent of LD was lower within Zebu and Sanga breeds compared to European *B. taurus*. They attributed the reduced extent of LD detected in these breeds to the SNP ascertainment bias that was due to the discovery of SNPs used in the design of this assay in European *B. taurus* breeds.

Success of LD applications across populations relies on the preservation of allele phase relationships between markers and QTL across populations (Hayes et al., 2009). Using SNP data, de Roos et al. (2008) showed that as two populations became genetically more diverged, the allele phase relationships were less likely to be conserved. Therefore, it is essential that the extent of LD and persistence of allelic phase relationships be estimated for populations in which GWAS, MAS and genomic selection are to be implemented. This information will be essential for identification of the optimal array to apply with regard to cost (number of genotyped individuals) and marker density (to achieve a satisfactory inter-marker LD) (Goddard and Hayes, 2007).

Extent of genome-wide LD has not yet been assessed in South African cattle breeds. The South African Sanga cattle breeds (Afrikaner, Nguni, Drakensberger, and Bonsmara) were previously described by Makina et al. (2014) and were shown to be genetically distinct from Angus and Holstein (European *B. taurus*). Sanga cattle are thought to be a hybrid of *B. taurus* and *B. indicus* origin which likely occurred as *B. taurus* Egyptian Longhorn cattle migrated south from Egypt and the Sudan, and *B. indicus* Lateral Horned Zebu cattle migrated from Arabia and India (Scholtz et al., 2011). Afrikaner, Nguni, and Drakensberger are indigenous breeds while the Bonsmara is a composite breed developed during the 1960s through the cross breeding of Afrikaner, Hereford, and Shorthorn (Bonsma, 1980). The four breeds included in this study were shown by Makina et al. (2014) to share some level of coancestry, but the breeds were clearly distinguishable from each other. Previous studies have shown that LD is population specific and can be heterogeneous between populations depending on the demographic histories of the populations (McKay et al., 2007; Thévenon et al., 2007; Edea et al., 2014). Thus, the aim of this study was to quantify the extent of genome-wide LD, determine the persistence of allele phase relationships and estimate effective population size for the South African Afrikaner, Nguni, Drakensberger, and Bonsmara Sanga cattle breeds using Angus and Holstein as reference groups since these breeds have previously been extensively characterized in other countries.

## Materials and methods

### Animal samples and quality control

The genotypic data for this study originated from the work by Makina et al. (2014) that included Afrikaner (*n* = 44); Nguni (*n* = 54), Drakensberger (*n* = 47), Bonsmara (*n* = 44), Angus (*n* = 31), and Holstein (*n* = 29). Two hundred and forty nine samples were selected based on pedigree data to select against full- and half-sib animals in order to maximize the genetic diversity within each sampled population. Blood and semen were used as sources of genomic DNA with approval of the Animal Ethics Committee of the University of Pretoria (E097-12). Genotypic data were generated using the Illumina BovineSNP50 BeadChip v2 (Matukumalli et al., 2009).

Quality control was performed within breed and included removing any SNPs with less than a 95% call rate, SNPs with minor allele frequency (MAF) less than 5% and SNPs which deviated significantly from Hardy-Weinberg Equilibrium (*P* < 0.001; Purcell et al., 2007). Only SNPs that were uniquely mapped to autosomes were included in the analyses. Samples with more than 10% missing genotypes and one sample of any pair with an identity by descent score of more than 0.25 were also excluded (Purcell et al., 2007). SNPs remaining after quality control are reported for each breed in Supplementary Material 1 and these were used for analyses. SNP Variation Suite (SVS) version 8.1 (SVS 8.1; Golden Helix Inc., Bozeman, Montana; Golden Helix Inc., 2012) was used to calculate the chromosome length (Mb) spanned by the retained markers, number of SNPs per chromosome and the average gap between SNPs in this study (Supplementary Material 2). Details of the physical positions of the markers used in this study were obtained from Illumina map files: (http://support.illumina.com/array/array_kits/bovinesnp50_v2_dna_analysis_kit/downloads.html).

### Minor allele frequency

The MAF for each breed was calculated using PLINK v1.07 under default settings (Purcell et al., 2007) for all autosomal SNPs. The distribution of allele frequencies was analyzed using *R* software (R Development Core Team, 2013) and proportions of SNPs in different frequency categories were plotted.

### Pattern of haplotype blocks

Haplotype blocks are defined as the particular combinations of alleles observed in a particular population for a genomic region in which less than 5% of comparisons among informative SNP pairs show strong evidence of historical recombination (Gabriel et al., 2002). Haplotype block based methods provide greater information content than single SNP methods in GWAS (Qanbari et al., 2010). In this study, the inference of haplotype was carried out directly from unphased genotypic data for each chromosome within each breed using the E-M algorithm approach implemented in PLINK v1.07 (Purcell et al., 2007). The direct phasing of genotypes across breeds was avoided, as this would likely harmonize the haplotypes into a set that would actually be less diverse than is correct due to the assumption that they all belong to a single panmictic population. PLINK v1.07 (Purcell et al., 2007) was used to estimate haplotype block structure, using default setting in haploview (http://www.broad.mit.edu/mpg/haploview/).

### Linkage disequilibrium analysis

To predict the extent of LD the r-squared statistic was chosen over the *D'* estimator. This choice allows for comparisons of results from this study with previous studies in cattle and other domestic animals. The *D'* estimate also tends to be inflated with small sample sizes or at low haplotype frequencies (McRae et al., 2002). The r-squared estimator is accepted as a measure of LD in the context of QTL mapping, due to applicability for estimation of the number of loci required for association studies (Pritchard and Przeworski, 2001). The *r*^2^ between syntenic pairwise SNPs separated by up to a distance of 1 Mb was used to estimate the extent of LD in PLINK v1.07 (Purcell et al., 2007) based on haplotype frequencies estimated via the E-M algorithm using the following parameters: –ld-snp-list mysnplist –ld-window-kb 10000 –ld-window 99999 –ld-window r2 0. LD was estimated genome-wide separately for each breed. The decay of LD was then analyzed for the following different genetic distance classes between SNP pairs [0, < 10 (10); 10, < 20 (20); 20, < 40 (40); 40, < 60 (60); 60, < 100 (100); 100, < 200 (200); 200, < 500 (500); 500, < 1000 (1000) kb]. To test the effects of MAF on the estimates of LD, the LD was calculated as previously described with different minimum MAF thresholds (MAF ≥ 0.05, MAF ≥ 0.1, MAF ≥ 0.2). *R* software (R Development Core Team, 2013) was used to visualize the effects of MAF on the genome-wide LD levels by plotting LD levels for the different breeds.

### Persistence of allele phase and time since breed divergence

Persistence of allele phase between the breeds can be used to determine the history and the relationships among breeds within a species. It can also be used to determine the reliability of MAS when performed across populations (Goddard et al., 2006). In this study, the persistence of phase between alleles on the same chromosome was estimated using *r*, (LD estimator is *r*^2^) for marker pairs that were in common (*n* = 21.869) amongst the six breeds using PLINK software version 1.07 (Purcell et al., 2007), with the following parameters: –r –ld-window-kb 1000 –ld-window 9999. The correlations among *r*-values between breeds were estimated for 10 kb intervals (from 0 to 1000 kb) using the PROC CORR procedure in SAS (SAS Institute Inc., Cary, USA).

The phase correlations were used to estimate the number of generations since the breeds diverged from a common ancestral population (Sved et al., 2008). When two populations have originated from a common ancestral population, their phase correlation can be expressed as $r_{0}^{2}{\left(1-\text{c}\right)}^{\text{2T}}$, where $r_{0}^{2}$ is a measure of LD in the common ancestral population, *c* is the recombination distance between markers, and *T* is time since breed divergence in generations (de Roos et al., 2008). The expected correlation of *r* between two breeds can be expressed as e^−2cT^ (de Roos et al., 2008). The recombination distance (*c*) is almost zero for markers separated by only 10 kb, thus the correlation of phase at these short distances (10 kb) serves as an approximation for the extent of LD in the common ancestral population (Badke et al., 2012). To estimate divergence time for South African cattle breeds, SNPs with pairwise distances between 0 and 500 kb in 10 kb intervals were used. A linear regression of the natural logarithm of the estimated correlation of *r* on average pairwise distance *c* was calculated. The slope of this regression gives −2*T*, which is divided by −2 to estimate *T* (de Roos et al., 2008).

### Effective population size

Effective population size (N_e_) of a real population X can be defined as the size of a hypothetical ideal population that will result in the same amount of genetic drift as in the (actual) population (Wright, 1931). It is an important population parameter that helps to explain how populations have evolved (Falconer and Mackay, 1996) and it can be used to improve understanding and modeling of the genetic architecture underlying complex traits (Hayes et al., 2003). In this study, the relationship between variance in LD and N_e_ was used to infer ancestral and recent effective population sizes and this was regarded as NeLD (Hayes et al., 2003; Tenesa et al., 2007). NeLD of a real population X with an observed LD for a given interval length was defined as the size of hypothetic ideal population that in an equilibrium state would display the same pattern of LD for the same interval length as observed in real finite population (Simianer, 2012). The relationship between LD and effective population size in the presence of mutation was estimated using the SNeP tool (Barbato et al., 2015) using the result of Corbin et al. (2012):

$$N_{T(T)}=(4f{\left(c_{\left(t\right)}\right)}^{-1}(E{\left[r_{\text{adj}}^{2}{}^{|\text{ct}}\right]}^{-1}-\text{ α})$$

Where *N*_*T*_ is the effective population size *T* generations ago calculated as *T* = ${\left(2f(c_{t})\right)}^{-1}$ (Hayes et al., 2003), *c*_*t*_ is the recombination rate for specific physical distance between markers calculated by SNeP tool (Barbato et al., 2015) using default values (1 Mb~1 cM), *r*^2adj^ = *r*^2^− (βn)^−1^ where *r*^2adj^ is the LD value adjusted for sample size (*n* is the sample size, β = 2 when gametic phase is known and β = 1 if unknown) and α is a correction for the occurrence of mutation.

## Results

### Marker statistics

Afrikaner had the smallest percentage of polymorphic SNPs remaining after data filtering (*n* = 30.484), while the Drakensberger and Holstein had the largest (*n* = 40.789 and 40,734, respectively; Supplementary Material 1). A summary of the SNP distribution by breed and chromosome is presented in Supplementary Material 2. The SNPs spanned approximately 2.49 Gb of the bovine autosomal genome. The distributions of SNPs varied amongst chromosomes with BTA1, the largest bovine autosome, containing the largest number of variable SNPs (2040–2674) after filtering. The lowest numbers of variable SNPs was observed on Chromosomes 25, 27, and 28; and BTA 27 and 28 in Afrikaner cattle, and Nguni respectively. In Drakensberger, Bonsmara, Angus, and Holstein the fewest variable SNPs was on BTA27. Chromosomes 25, 27, and 28 are among the smaller chromosomes and therefore expected that these would have fewer variable SNPs. The greatest average physical distance between SNPs was observed for Afrikaner (81.65 kb) whilst the smallest average inter-marker interval was observed in Drakensberger (61.09 kb) and Holstein (61.08 kb).

### Minor allele frequency

**Figure 1:**
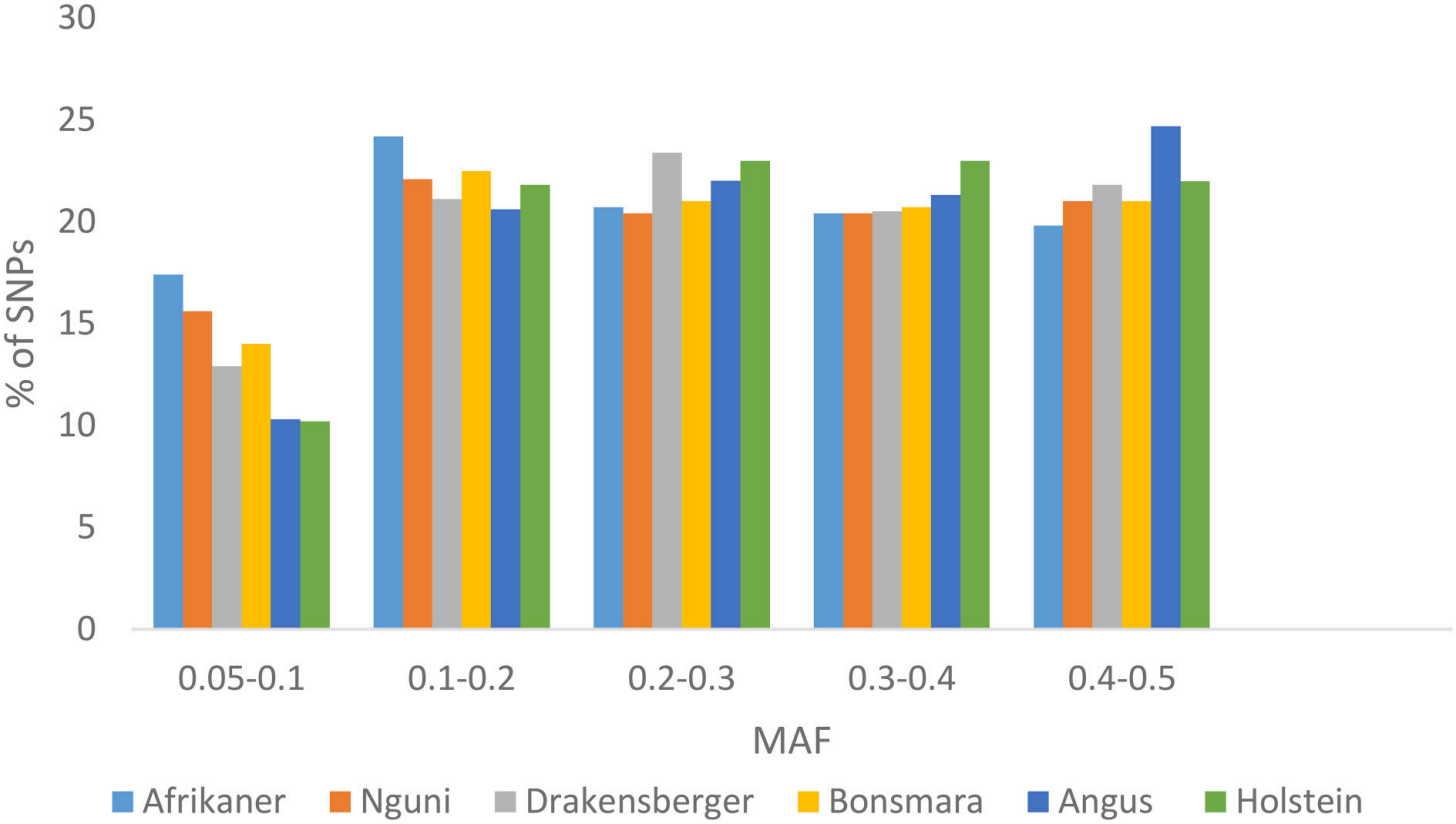
**Minor allele frequencies for SNPs that passed quality control by breed**.

The average MAF over all chromosomes was 0.25 ± 0.13 (Afrikaner), 0.26 ± 0.13 (Nguni), 0.27 ± 0.13 (Drakensberger), 0.26 ± 0.13 (Bonsmara), 0.28 ± 0.12 (Angus), and 0.28 ± 0.13 (Holstein). The distribution of MAF for these breeds is presented in Figure 1. Afrikaner and Nguni cattle had the highest percentage of SNPs with MAF in the range 0.05–0.1, while Holstein and Angus had the lowest percentage in this range.

### Haplotype block structure

**Figure 2:**
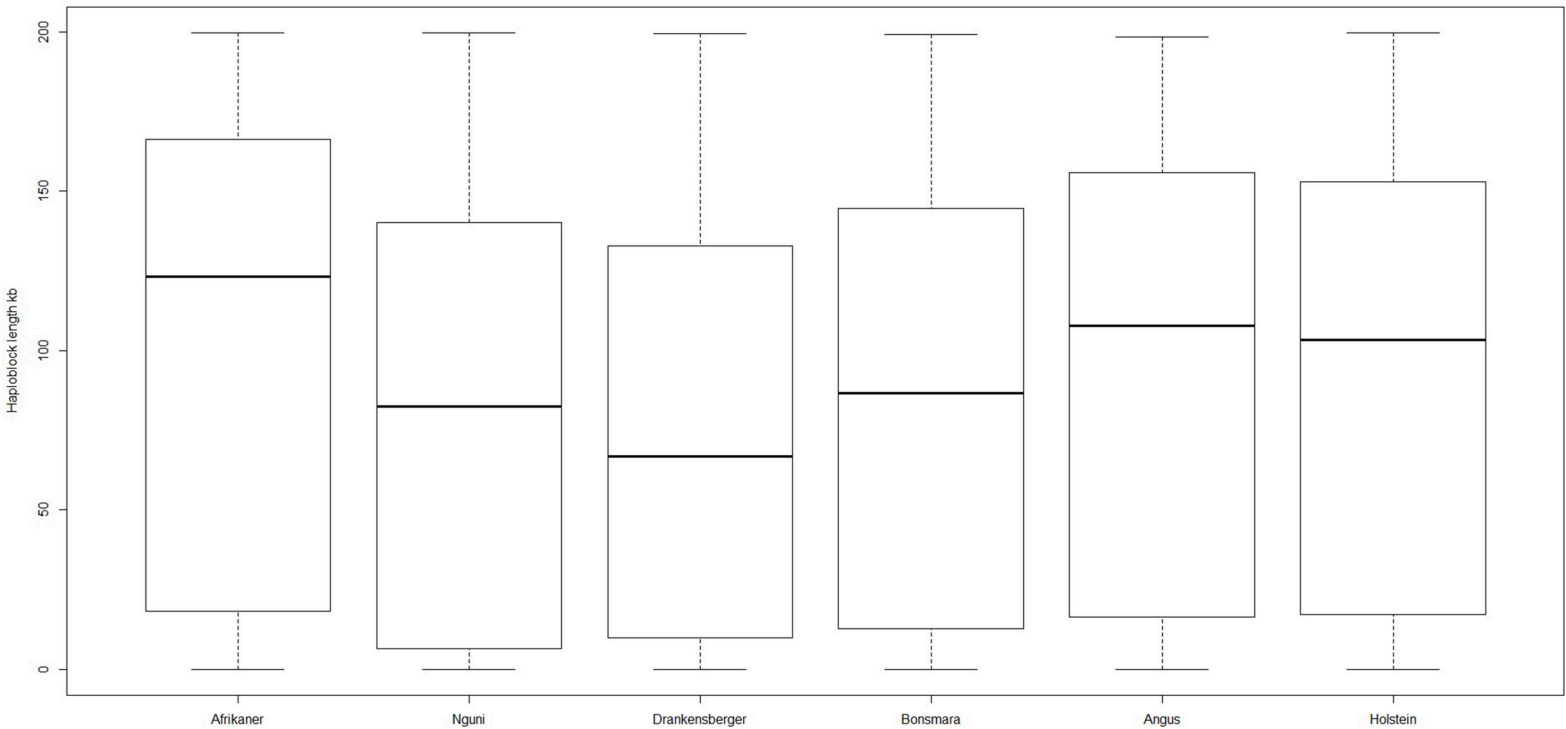
**Box plot of haploblock size in different breeds**.

The genome-wide haplotype block distribution across the studied breeds is presented in Table 1. A total of 320, 223, 285, 314, 446, and 452 haplotype blocks spanning between 0.65 and 1.67 Mb of the genome were detected in Afrikaner, Nguni, Drakensberger, Bonsmara, Angus, and Holstein, respectively. The mean number of SNPs contained within the haplotype blocks ranged from 3.30 (Nguni) to 3.73 (Afrikaner). The distribution of haplotype block size per breed is shown in Figure 2. Afrikaner cattle had the longest haplotype blocks and Drakensberger had the shortest.

**Table 1:** **Summary statistics for haploblock structure across breeds**.

	**Afrikaner**	**Nguni**	**Drakensberger**	**Bonsmara**	**Angus**	**Holstein**
Blocks (*n*)	320	223	285	314	446	452
Total block length^a^ (Mb)	33.33	16.77	21.11	25.78	42.33	42.53
Mean block length (kb)	104.16	75.18	74.05	82.12	94.90	94.09
SNP in blocks	1193	736	953	1098	1700	1685
Mean number of SNPs in blocks	3.73	3.30	3.34	3.50	3.8	3.72
Max number of SNPs in blocks	7	6	7	7	8	8

a *Cumulative length of all detected haplotype blocks*.

### Linkage disequilibrium decay

**Figure 3:**
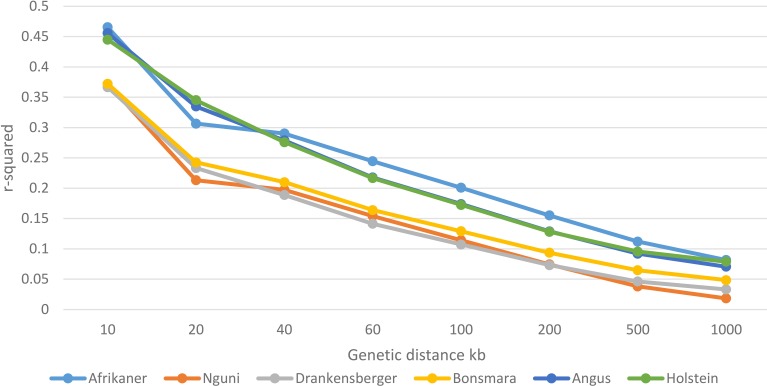
**Decay of LD by distance across the studied breeds**.

Genome-wide average LD (*r*^2^) decreased with increasing genomic distance for all breeds (Figure 3). Large differences between the Afrikaner cattle and other local breeds (Nguni, Drakensberger, and Bonsmara) were observed in this study. Nguni, Drakensberger, and Bonsmara had lower LD, which rapidly decayed with increasing distance between markers compared to the Afrikaner. As expected the Angus and Holstein cattle had higher LD compared to local breeds with the exception of the Afrikaner.

### MAF and LD

**Figure 4:**
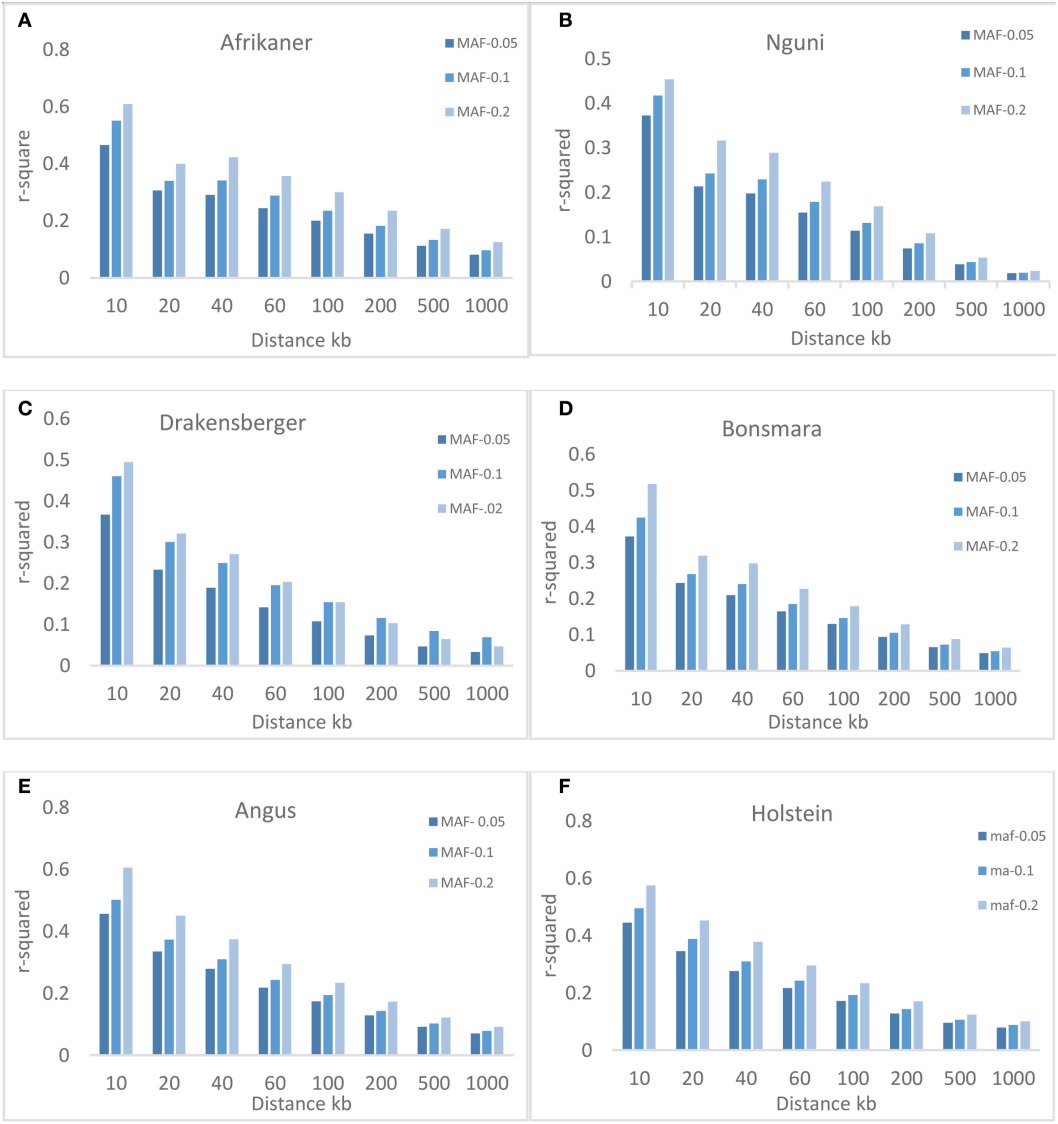
**Average *r*^2^ estimates at different genetic distances for three different minor allele frequency (MAF) thresholds**. Average LD estimates are pooled over all autosomal chromosomes: **(A)** Afrikaner, **(B)** Nguni, **(C)** Drakensberger, **(D)** Bonsmara, **(E)** Angus and, **(F)** Holstein.

Three different minimum MAF thresholds (0.05, 0.1, and 0.2) were used to study the effect of MAF on the extent of LD. Figure 4 shows that MAF had an effect on the average value of *r*^2^, especially over short inter-marker distances (< 100 kb). The average *r*^2^ increased with MAF, for example, for markers with MAF ≥ 0.05 and separated by 0–10 kb, the average *r*^2^ was 0.47, 0.37, 0.37, 0.37, 0.46, and 0.45. For markers with MAF ≥ 0.1, the *r*^2^ estimates increased to 0.55, 0.42, 0.46, 0.42, 0.50, and 0.50; these estimates further increased to 0.61, 0.45, 0.49, 0.52, 0.60, and 0.57 with MAF ≥ 0.2, for Afrikaner, Nguni, Drakensberger, Bonsmara, Angus, and Holstein, respectively.

### Persistence of phase and time since breed divergence

**Figure 5:**
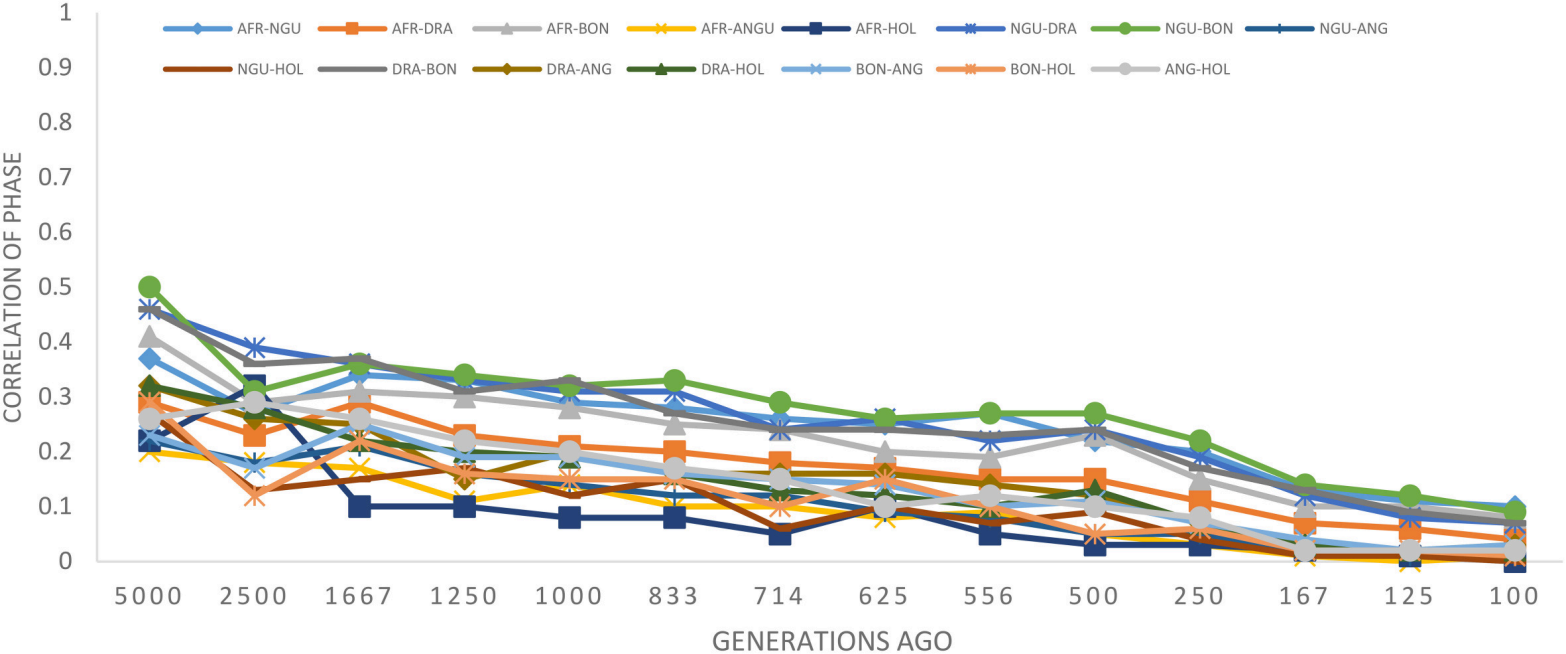
**Correlation of allele phase by physical distance represented as generations in the past**.

**Figure 6:**
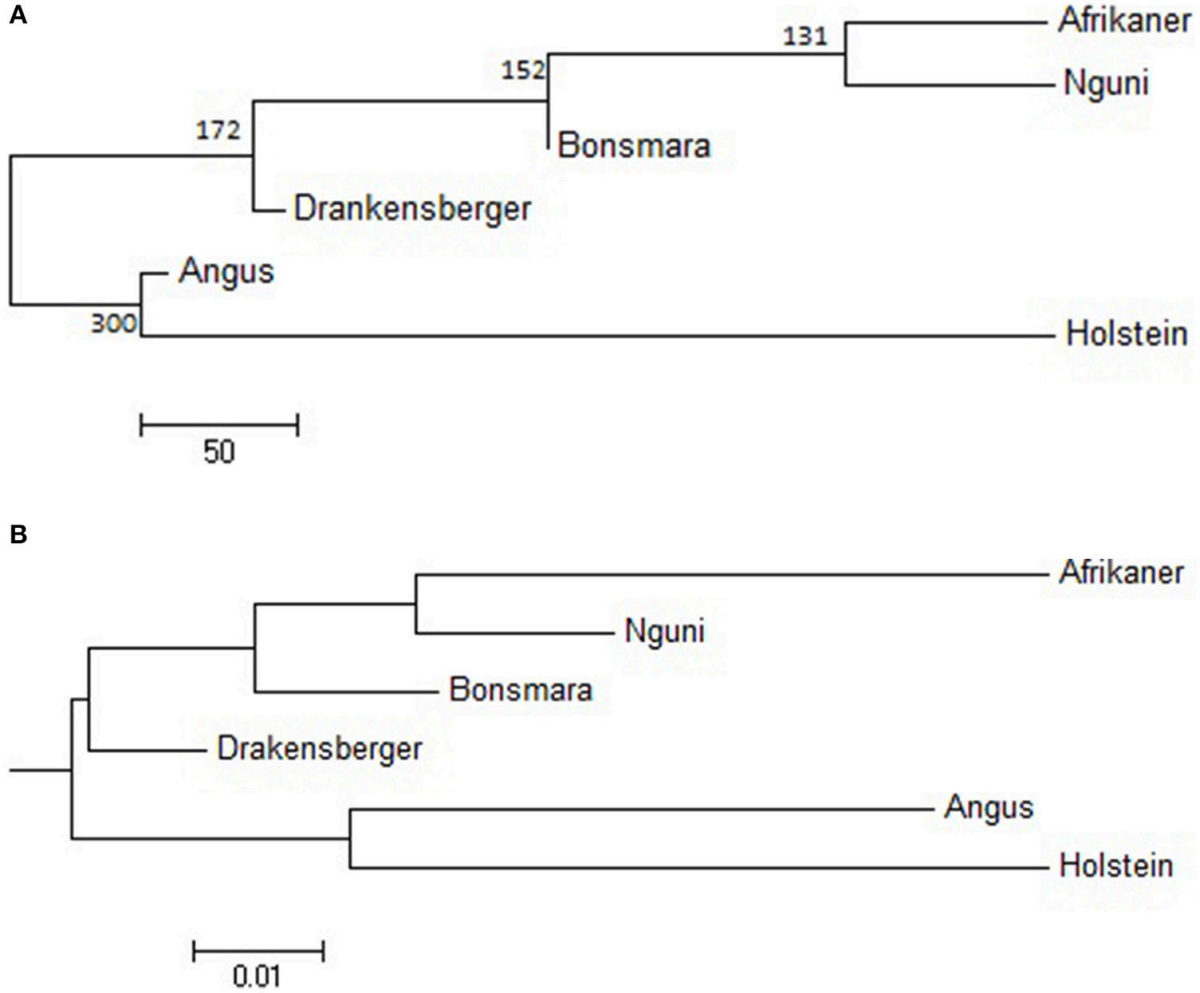
**(A)** Neighbor-joining tree showing time since breed divergence in generations, **(B)** Neighbor-joining tree showing the genetic relationship among breeds based on *F*_*ST*_.

The statistic *r* was used to examine the extent of persistence of marker allele phase in the studied breeds. For example, if *r* was positive in one population and negative in another population, then different haplotypes are prevalent in the two populations and phase is not preserved between them. However, if *r* is positive in both populations, then similar haplotypes are common in both populations and phase tends to be preserved. A correlation of 1 between *r* values indicates that the marker phase persists across populations while a correlation of zero indicates that the marker phase in population one cannot predict phase in the other population (de Roos et al., 2008). In this study, the correlation between alleles at contiguous loci indicated that phase was not strongly preserved between breeds and decreased with increasing genetic distance between the breeds (Figure 5). The largest correlation was observed between the Nguni and Bonsmara pair (0.50 at 10 kb) whilst the lowest correlation was observed between Afrikaner and Drakensberger (0.29 at 10 kb). As expected, the correlations between *r* statistics for all *B. taurus*-African breed pairs were lower than those found among the South African breeds (Figure 5) with the lowest correlation observed between Afrikaner and Angus for marker pairs separated by less than 10 kb. This was supported by the estimated time of divergence of these breeds that suggested that South African cattle breeds diverged from each other approximately 131 (Nguni vs. Afrikaner) to 192 (Afrikaner vs. Drakensberger) generations ago (Supplementary Material 3). On the other hand *B. taurus* breeds diverged from the South African cattle breeds approximately 245 (Bonsmara vs. Angus) to 884 generations ago (Nguni and Holstein). Figure 6 shows the neighbor-joining tree based on time since breed divergence and also a neighbor-joining tree based on the *F*_*st*_-values reported by Makina et al. (2014).

### Effective population size

**Figure 7:**
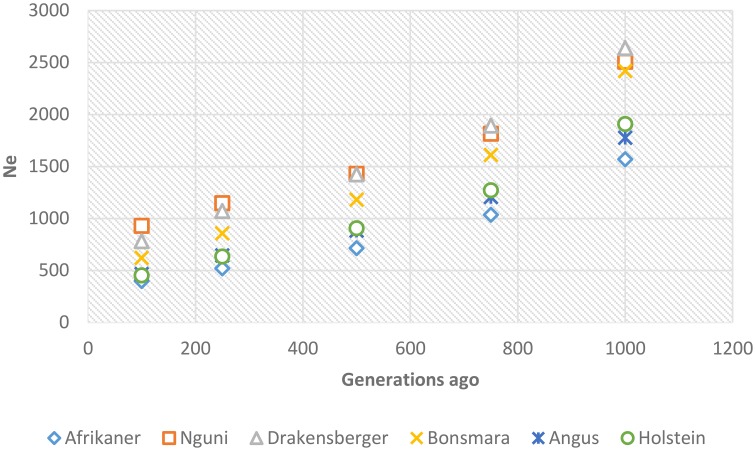
**Genome-wide estimates of historical effective population size (N_e_) over the previous 1000–100 generations based on estimates of linkage disequilibrium**.

**Figure 8:**
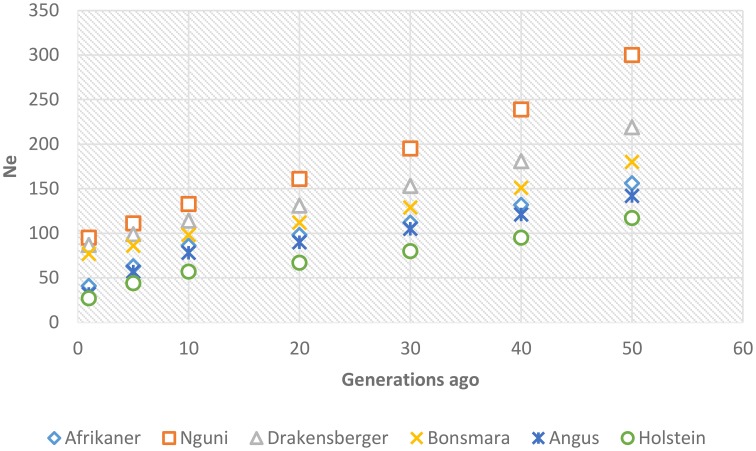
**Genome-wide estimates of recent effective population size (N_e_) from 1–50 generations in the past based on estimates of linkage disequilibrium**.

Past and recent effective population sizes were estimated from the average *r*^2^ for markers separated by various genomic distances. The extent of LD over greater recombinational distances indicated more recent N_e_ while that over shorter distances provided ancestral N_e_ (Hayes et al., 2003). Figure 7 presents historical N_e_, i.e., from 1000 to 100 generations ago while Figure 8 shows more recent N_e_. In general, N_e_ declined over time from larger to smaller N_e_ across the breeds. Nguni, Drakensberger, and Bonsmara had greater estimates of effective population size than did the Afrikaner, Angus, and Holstein at all generations.

## Discussion

This study utilized the BovineSNP50 genotyping assay to estimate the extent of LD, N_e_, and the persistence of phase among cattle breeds in South Africa. As previously reported by Qwabe et al. (2013), South African cattle breeds with the exception of Drakensberger cattle had fewer polymorphic loci and a higher percentage of SNPs with low MAF (MAF < 0.05) compared to the European taurine breeds (Angus and Holstein). The lower percentage of polymorphic loci among South African cattle breeds has previously been attributed to the ascertainment bias associated with the design of the BovineSNP50 BeadChip (Qwabe et al., 2013), as the SNP used in the design of this assay were detected in European *B. taurus* breeds, resulting in the MAF being lower in *B. indicus* breeds. SNPs with low allele frequencies tend to underestimate *r*^2^ estimates of LD between markers (Khatkar et al., 2008; Bohmanova et al., 2010; Qanbari et al., 2010; Espigolan et al., 2013). Three different MAF thresholds (0.05, 0.1, and 0.2) were used to study the effect of MAF on the extent of LD (Figure 4) and LD was found to increase as MAF increased across all breeds, especially for SNP pairs separated by short distances (< 100 kb). This phenomenon was previously observed by Khatkar et al. (2008) in Australian Holstein-Friesian cattle. They found that the mean *r*^2^ was 0.59 for SNP with MAF ≥ 0.05 but was higher (0.70) for SNP with MAF ≥ 0.2 separated by 1–10 kb. They attributed this to the fact that as the MAF threshold increases, there is an increase in the number of SNP pairs with similar allele frequencies and therefore an increase in *r*^2^ (Khatkar et al., 2008). On the other hand, Bohmanova et al. (2010) showed that the D' parameter was the highest for SNP with low MAF and thus overestimated LD among these loci while LD was underestimated for SNP with higher MAF. A study by Qanbari et al. (2010) reported that by minimizing the allele frequency difference between SNPs a more sensitive and useful metric for characterizing LD was achieved. In another study, using the BovineHD Chip on European taurine and Zebu cattle, O'Brien et al. (2014) observed no evidence of an influence of MAF on *r*^2^ estimates. They concluded that unbiased estimates of LD were obtained provided that MAF > 0.05 unless low density SNP coverage assays were used.

Small sample size (*n* ≤ 25) can also lead to biased estimates of LD (Khatkar et al., 2008). However, the results of Bohmanova et al. (2010) illustrated that sample size did not have a large effect on *r*^2^-values when at least 55 samples were used for the calculation. In this study, the results for Holstein and Angus cattle were comparable to previous studies employing larger sample sizes (>100; Khatkar et al., 2008; Bohmanova et al., 2010; Lu et al., 2012). However, the interpretation of these results should be performed with caution, as the establishment of the true LD values will require large sample sizes of unrelated individuals. Thus, future assessments of LD for South African Sanga cattle using the BovineHD Chip or whole genome sequence data and larger samples may address any biases that might have been introduced in this study due to sample size or ascertainment bias.

In general, the Angus and Holstein had more haplotype blocks than did the South African cattle breeds. Gautier et al. (2007) observed a similar trend for West African cattle breeds. This is likely due to the ascertainment bias that is associated with the design of the SNP assays. The number of haplotype blocks reported for Holstein (452) in this study was fewer than that reported by Qanbari et al. (2010) in German Holstein (712) and by Khatkar et al. (2008) in Australian Holstein-Friesian cattle (727). However, this was higher than the number reported by Gautier et al. (2007) in Holstein (94). The observed differences may be ascribed to the different marker densities used between these studies again due to ascertainment bias in assay design. The mean block size varied from 74 (Drakensberger) to 104 kb (Afrikaner) and an average of 3.30–3.73 markers per block was observed across the breeds. These results are comparable to those reported by Villa-Angulo et al. (2009) in cattle, who reported haplotype block ranges of between 30 and 75 kb and average markers of 3.8 in blocks. Haplotypic methods have been suggested to be more powerful for detecting genetic variation responsible for phenotypes (Villa-Angulo et al., 2009), because they provide more information to estimate whether two alleles are identical by descent; they reduce the number of association tests performed and hence the type I error rate, allow informed testing between clades of haplotype alleles by capturing evolutionary information and provide more power than single SNPs when multiple alleles exist at a causal locus (Gautier et al., 2007; Villa-Angulo et al., 2009; Qanbari et al., 2010).

The estimate of LD for SNP pairs separated by 40–60 kb was lower in the Nguni (0.15), Drakensberger (0.14), and Bonsmara (0.16) compared to the Afrikaner (0.24), Angus (0.21), and Holstein (0.21) which were similar to estimates reported for Angus and Holstein cattle located in other regions of the world (McKay et al., 2007; Khatkar et al., 2008; Lu et al., 2012). Low levels of LD have previously been reported by Edea et al. (2014) for Ethiopian cattle (0.14) and by Espigolan et al. (2013) in Nellore cattle (0.17) at a similar genetic distance. Low levels of LD among indicus, Zebu, and Sanga breeds have previously been attributed to the ascertainment bias of the SNPs located on the BovineSNP50 assay (Edea et al., 2014). In addition, the removal of many SNPs with very low MAF was previously found to be associated with biased estimates of LD (Khatkar et al., 2008; Espigolan et al., 2013). However, in this study, the Afrikaner cattle which had about 20% less variable SNPs and a higher proportion of low MAF SNPs compared to Drakensberger, Angus, and Holstein cattle, had higher levels of LD at all genetic distances. Consequently, the differences in the estimates of LD generated in this study can be ascribed to different evolutionary and molecular forces that have acted differently on these cattle breeds (Lee et al., 2011). Decker et al. (2012) showed that at least 80% of the US Angus genome was under strong selection, thus selection could be considered to be one of the causes of LD in livestock. Small effective population size is also usually implicated as the key source of extensive LD in livestock populations (Hayes et al., 2003). This is likely to be the situation for the Afrikaner cattle as in the recent past, this breed has significantly declined in popularity and in numbers due to high levels of inbreeding and reduced fertility (Coetzer and van Marle, 1972; Pienaar et al., 2014). The Nguni, Drakensberger, and Bonsmara reveal similar declines in LD, probably because these breeds share some ancestry (Makina et al., 2014) and are similar in effective population sizes and history.

Persistence of allele phase was used to infer the history and the genetic relationships among breeds based on their extent of divergence (de Roos et al., 2008). The correlation between alleles at contiguous loci indicated that phase was not strongly preserved between breeds. This suggests the need for breed-specific reference populations in which a much greater density of markers should be scored to identify breed specific haplotypes which may then be imputed into multi-breed commercial populations. The observed correlation of allele phases between the breeds in this study was much lower than previously reported by de Roos et al. (2008) for *B. taurus* breeds and by Gautier et al. (2007) between European cattle breeds at a short range (<10 kb).

Persistency of phase was used to estimate the time since breed divergence using the method proposed by de Roos et al. (2008). Results for Angus and Holstein (*T* = 300 generations ago) were in agreement with those reported by de Roos et al. (2008) for these breeds. In addition, our results suggested that the South African cattle breeds diverged approximately 131–192 generations ago, with Afrikaner and Nguni (131) having the lowest divergence time. This is in agreement with the recorded history of these breeds; Afrikaner and Nguni cattle are both Sanga breeds and are believed to have been derived from a common ancestor, a cross-bred between *B. indicus* and *B. taurus* cattle, which was assumed to originate from cattle that migrated south from Egypt and Sudan, and cattle that migrated from Arabia and India (Scholtz et al., 2011). Angus and Holstein are diverged from South African cattle breeds by approximately 245 and 900 generations, respectively, with the Angus having a considerably smaller divergence time than Holstein. This was in agreement with the previous estimates of divergence times between European *B. taurus* and African cattle (McKay et al., 2008).

LD structure can be used to provide insights into the evolutionary history of a population (Hill, 1981) and in this study the strength of LD at different genetic distances between loci was used to estimate ancestral effective population sizes. We found a decline in N_e_ throughout time in all breeds; between about 1000 generations until 100 generations ago, N_e_ declined across the breeds. This decrease in N_e_ may be associated with the post-domestication events of human migration with cattle that ultimately led to breed formation (Gautier et al., 2007). The most rapid decline in Ne occurred over the last 100 generations (approximately 500 years ago until present—assuming a generation interval of 5 years) in all breeds. This may suggest that a significant bottleneck occurred at breed formation and that population subdivision resulted in significantly reduced Ne (Daetwyler et al., 2010). Nguni, Drakensberger and Bonsmara had greater effective population sizes at all generations compared to Afrikaner probably due to presence of admixture within these breeds (Makina et al., 2014). de Roos et al. (2008) estimated effective population size in Australian Holstein-Friesian, Jersey and Angus cattle and found that the Ne for these breeds has decreased over the last 50 generations to approximately 100. Similarly, Villa-Angulo et al. (2009) estimated Ne for US Angus and found a rapid decline in N_e_ in Angus over the last 100 generations. In addition, Decker et al. (2012) estimated Ne in North American Angus using molecular inbreeding coefficients and found current Ne to be 94. On the other hand, effective population sizes obtained for Afrikaner, Nguni, and Bonsmara cattle in this study were in agreement with those reported based on pedigree data for the Afrikaner (Pienaar et al., 2014), Nguni (Matjuda, 2012), and Bonsmara (Santana et al., 2015). The low effective population sizes for the Angus and Holstein breeds compared to those for the Nguni, Bonsmara, and Drakensberger at more recent generations, could be due to intense selection and probably widespread use of artificial insemination and the use of relatively few elite sires after 1970 (Hayes et al., 2009). On the other hand, the low effective population size observed in the Afrikaner cattle could be associated with the bottleneck history of this breed (Coetzer and van Marle, 1972; Pienaar et al., 2014).

### Implications for QTL mapping and genomic selection

The extent and patterning of LD within the breeds represented in this study was used to assess the number of markers that would be required for GWAS of the six cattle breeds in South Africa. LD can be defined as the *r*^2^ between a marker and a QTL and this estimator is the proportion of QTL variance that can be observed at the marker (Hayes et al., 2009). Thus, the threshold for useful LD was assumed to be *r*^2^≥ 0.20 in this study as proposed by Hayes et al. (2003) for the application of genomic selection and GWAS. We found that for the Holstein and Angus cattle breeds, SNP spacing should be approximately 40–60 kb for genome-wide association scans. Assuming that any QTL will be at most in the middle of the interval, and therefore no more than 30 kb away from any marker, a minimum of 50,000 evenly spaced and informative markers would be sufficient to enable genome-wide scans in these breeds. This agrees with McKay et al. (2007) who suggested that 50,000 SNPs would capture most of the LD information necessary for GWAS in European *B. taurus* cattle populations. On the other hand, useful LD extended up to 100 kb in the Afrikaner cattle suggesting that approximately 30,000 uniformly distributed SNPs would be necessary in this breed in agreement with the findings of Villa-Angulo et al. (2009). However, for the Nguni and Drakensberger, the average *r*^2^ of 0.23 and 0.21 was achieved for inter-marker distances of 10–20 kb. This suggests that there should be an informative SNP every 20 kb to achieve the same GWAS power as in Afrikaner, Holstein and Angus, indicating that about 150,000 SNPs would be required in these breeds for genome-wide association and genomic selection studies, while 75,000 SNPs should be sufficient in Bonsmara. These results agree with Gautier et al. (2007) and Khatkar et al. (2008) who suggested that 75,000–300,000 informative SNPs would capture most of the LD information within the world's cattle breeds.

Persistence of allele phase between breeds in this study was used to determine the marker density to conduct multi-breed genomic selection. Correlation between alleles at contiguous loci indicated that phase was not strongly preserved between breeds. For example if a QTL was found in a chromosomal region in Nguni, SNPs linked to the QTL will only have a 50% chance of predicting the same QTL alleles in the Bonsmara breed given that region has an LD of at least 0.2 (Hayes et al., 2009). This suggests that there is considerable diversity amongst South African cattle breeds, and to find markers that consistently predict QTL alleles, i.e., with an *r* correlation >0.8 (de Roos et al., 2008), across all South African breeds of cattle, the marker to QTL interval should be less than 5 kb, which corresponds to the need to assay approximately 560,000 markers evenly distributed throughout the genome. This compares favorably with the studies of Goddard et al. (2006), de Roos et al. (2008), and Villa-Angulo et al. (2009) who indicated the value of having a large number of SNPs to cover the genome for genomic selection when analyzing data from multi-breed populations. In general, these results suggest that genomic based improvement programs for South African cattle breeds should consider using a much higher density SNP panel such as the Illumina BovineHD assay with 770 K SNPs and breed-specific reference populations to establish haplotypes or include purebred representatives from each breed in the reference population if a multi-breed GWAS is performed.

## Conclusions

This study revealed significant differences in the extent of LD between South African breeds. Afrikaner cattle had the highest levels of LD compared to the other indigenous breeds. The higher LD suggests that Afrikaner cattle have experienced considerable bottlenecks restricting their effective population size in contrast to the other indigenous breeds. This result also indicates that this breed would require lower marker density panels relative to those required for the Nguni, Drakensberger, and Bonsmara cattle to associate genetic variation with economically important traits. Furthermore, our results suggest the necessity of much higher SNP density (e.g., 770 K) panels and breed-specific reference populations or adequate representation of each breed in the training population if a multi-breed training population is to be used for genomic improvement programs.

## Author contributions

SM collected the genetic materials, carried out the laboratory analyses, statistical analyses, and interpretation of the data and drafted the manuscript. SM, FM, and AM assisted with the acquisition of funding. All authors participated in the design and coordination of the study. JT and MLM assisted with the statistical analyses. JT, EV, FM, MLM, MDM, and AM revised the manuscript critically for important scientific content. All authors read and approved the final manuscript.

### Conflict of interest statement

The authors declare that the research was conducted in the absence of any commercial or financial relationships that could be construed as a potential conflict of interest.
