# Supplementary material for: Extent of Linkage Disequilibrium and Effective Population Size in Four South African Sanga Cattle Breeds
Source: Front Genet. 2015 Dec 1;6:337. doi: 10.3389/fgene.2015.00337 (PMC4664654; doi:10.3389/fgene.2015.00337)
Supplement: Supplementary file 1 [file DataSheet1.DOCX]

***Supplementary Material***

**Extent of linkage disequilibrium and effective population size in four South African Sanga cattle breeds**

**S. O. Makina^1, 2*^, J. F. Taylor^3^, E. van Marle-Köster^2^, F. C. Muchadeyi^4^, M. L. Makgahlela^1^, M. D. MacNeil^1, 5, 6^ & A. Maiwashe^1, 5^**

^1^Agricultural Research Council-Animal Production Institute, Private Bag X 2, Irene 0062, South Africa

^2^Department of Animal and Wildlife Sciences, University of Pretoria, Private Bag X 20, Hatfield 0028, South Africa

^3^Division of Animal Sciences, University of Missouri, Columbia, MO 65211, USA

^4^Agricultural Research Council-Biotechnology Platform, Private Bag X 5, Onderstepoort, 0110, South Africa

^5^Department of Animal, Wildlife and Grassland Sciences, University of Free State, Bloemfontein 9300, South Africa

^6^Delta G, Miles City, MT 59301, USA

*Correspondence:

Dr. Sithembile Makina

Private Bag X 2

Irene

0062

South Africa

[qwabes@arc.agric.za](mailto:qwabes@arc.agric.za)

**Supplementary Material 1 (SM1):** Number of SNPs genotyped and remaining after quality control filters.

| **Breeds** |  |  | **SNPs removed** |  | **Final SNPs** |  |  |  |
| --- | --- | --- | --- | --- | --- | --- | --- | --- |

|  | **Call rate < 95%** | **HWE**  **(p < 0.001)** | **MAF**  **<0.05** | **Sex & *unmapped** | **Polymorphic SNP (used)** | **% Polymorphic** | ***Average Gap**  **(kb)** |
| --- | --- | --- | --- | --- | --- | --- | --- |
| Afrikaner | 725 | 279 | 22623 | 911 | 30484 | 56 | 81.65 |
| Nguni | 2191 | 237 | 16436 | 963 | 35479 | 65 | 70.08 |
| Drakensberger | 925 | 327 | 11825 | 118 | 40789 | 74 | 61.09 |
| Bonsmara | 1871 | 140 | 12862 | 1018 | 39215 | 72 | 63.65 |
| Angus | 916 | 198 | 12787 | 1135 | 39831 | 73 | 62.44 |
| Holstein | 646 | 121 | 12127 | 1161 | 40734 | 74 | 61.08 |

***SNP position from the UMD 3.1 assembly.**

**Supplementary Material 2 (SM2):** Summary of SNP distribution by chromosome and breed

|  |  | **Afrikaner** |  | **Nguni** |  | **Drakensberger** |  | **Bonsmara** |  | **Angus** |  | **Holstein** |  |
| --- | --- | --- | --- | --- | --- | --- | --- | --- | --- | --- | --- | --- | --- |
| **Chromosome** | **Length (Mb)** | **Number of SNP** | **Average**  **spacing (kb)** | **Number of SNP** | **Average spacing (kb)** | **Number of SNP** | **Average spacing (kb)** | **Number of SNP** | **Average spacing (kb)** | **Number of SNP** | **Average spacing (kb)** | **Number of SNP** | **Average spacing (kb)** |
| 1 | 158.03 | 2040 | 77.46 | 2287 | 69.1 | 2646 | 59.72 | 2562 | 61.71 | 2542 | 62.19 | 2674 | 59.1 |
| 2 | 136.66 | 1684 | 81.15 | 1929 | 70.83 | 2196 | 62.23 | 2155 | 63.42 | 2146 | 63.68 | 2118 | 64.52 |
| 3 | 121.14 | 1469 | 82.47 | 1762 | 68.75 | 2033 | 59.59 | 1880 | 64.44 | 1945 | 62.28 | 1987 | 60.97 |
| 4 | 120.36 | 1515 | 79.45 | 1687 | 71.35 | 1960 | 61.43 | 1892 | 63.62 | 1898 | 63.46 | 1932 | 62.44 |
| 5 | 121.08 | 1235 | 98.04 | 1467 | 82.53 | 1698 | 71.31 | 1657 | 73.07 | 1599 | 75.72 | 1738 | 69.67 |
| 6 | 119.05 | 1529 | 77.86 | 1749 | 67.98 | 1973 | 60.32 | 1937 | 61.44 | 1984 | 59.99 | 2043 | 58.27 |
| 7 | 112.27 | 1295 | 86.7 | 1508 | 74.53 | 1773 | 63.39 | 1733 | 64.85 | 1731 | 64.92 | 1753 | 64.06 |
| 8 | 112.91 | 1373 | 82.23 | 1622 | 69.61 | 1879 | 60.09 | 1818 | 62.11 | 1850 | 61.03 | 1873 | 60.28 |
| 9 | 105.46 | 1215 | 86.8 | 1345 | 78.41 | 1602 | 65.83 | 1511 | 69.8 | 1571 | 67.13 | 1596 | 66.08 |
| 10 | 104.17 | 1277 | 81.58 | 1518 | 68.63 | 1723 | 59.88 | 1695 | 61.46 | 1701 | 60.66 | 1693 | 61.53 |
| 11 | 107.14 | 1227 | 87.32 | 1546 | 69.3 | 1766 | 60.69 | 1715 | 62.47 | 1710 | 62.65 | 1771 | 60.52 |
| 12 | 90.82 | 922 | 98.5 | 1190 | 76.32 | 1341 | 67.82 | 1283 | 70.88 | 1304 | 69.74 | 1334 | 68.09 |
| 13 | 83.84 | 1004 | 83.5 | 1179 | 71.11 | 1433 | 58.52 | 1336 | 62.77 | 1377 | 60.9 | 1385 | 60.39 |
| 14 | 83.15 | 1100 | 75.59 | 1263 | 65.84 | 1431 | 58.11 | 1401 | 59.35 | 1372 | 60.61 | 1426 | 58.31 |
| 15 | 84.22 | 1034 | 81.45 | 1176 | 71.62 | 1349 | 62.43 | 1274 | 65.95 | 1279 | 65.69 | 1388 | 60.84 |
| 16 | 81.25 | 985 | 82.49 | 1156 | 70.28 | 1294 | 62.79 | 1261 | 64.36 | 1276 | 63.68 | 1275 | 63.73 |
| 17 | 74.78 | 905 | 82.63 | 1074 | 69.73 | 1254 | 59.72 | 1188 | 63.04 | 1228 | 60.96 | 1278 | 58.6 |
| 18 | 65.4 | 807 | 81.04 | 917 | 70.98 | 1053 | 61.88 | 990 | 65.82 | 1054 | 62.05 | 1068 | 61.24 |
| 19 | 63.51 | 819 | 77.55 | 939 | 67.64 | 1115 | 56.99 | 1031 | 61.63 | 1097 | 57.92 | 1123 | 56.58 |
| 20 | 71.59 | 954 | 75.05 | 1071 | 66.85 | 1252 | 57.18 | 1196 | 59.86 | 1222 | 58.44 | 1237 | 57.88 |
| 21 | 69.45 | 858 | 80.95 | 982 | 72.4 | 1130 | 62.92 | 1084 | 65.59 | 1086 | 65.47 | 1111 | 63.97 |
| 22 | 61.22 | 735 | 83.29 | 928 | 65.97 | 1031 | 59.38 | 993 | 61.65 | 992 | 61.71 | 1005 | 60.79 |
| 23 | 52.1 | 704 | 74 | 765 | 68.1 | 853 | 61.23 | 807 | 64.72 | 848 | 61.59 | 846 | 61.73 |
| 24 | 62.05 | 747 | 83.07 | 893 | 69.51 | 1019 | 60.94 | 993 | 62.54 | 1001 | 62.08 | 1011 | 61.47 |
| 25 | 42.62 | 567 | 75.17 | 688 | 62.13 | 752 | 56.92 | 767 | 55.81 | 768 | 55.73 | 810 | 52.84 |
| 26 | 51.58 | 673 | 76.65 | 770 | 66.29 | 865 | 59.63 | 842 | 60.62 | 855 | 59.59 | 857 | 59.46 |
| 27 | 45.25 | 579 | 78.15 | 665 | 68.05 | 750 | 60.44 | 708 | 64.03 | 741 | 61.18 | 770 | 58.87 |
| 28 | 46.18 | 578 | 79.9 | 654 | 70.62 | 773 | 59.75 | 723 | 63.88 | 771 | 59.9 | 780 | 59.21 |
| 29 | 50.83 | 654 | 77.72 | 749 | 67.89 | 845 | 60.48 | 783 | 64.97 | 853 | 59.91 | 852 | 59.98 |
| All | 2498.13 | 30484 | 81.65 | 35479 | 70.08 | 40789 | 61.09 | 39215 | 63.65 | 39801 | 62.44 | 40734 | 61.08 |

**Supplementary Material 3 (SM3):** Estimates of time since breed divergence

| Breed pairs^1^ | Divergence time (generations) |
| --- | --- |
| AFR vs NGU | 131.64 |
| AFR vs DRA | 192.35 |
| AFR vs BON | 151.31 |
| AFR vs ANG | 345.31 |
| AFR vs HOL | 840.45 |
| NGU vs DRA | 182.15 |
| NGU vs BON | 152.09 |
| NGU vs ANG | 333.03 |
| NGU vs HOL | 884.17 |
| DRA vs BON | 172.78 |
| DRA vs ANG | 269.98 |
| DRA vs HOL | 289.57 |
| BON vs ANG | 245.04 |
| BON vs HOL | 309.10 |
| ANG vs HOL | 300.86 |

^1^Afrikaner (AFR), Drakensburg (DRA), Nguni (NGU), Bonsmara (BON), Angus (ANG).
